# Serovar Diversity of Pathogenic *Leptospira* Circulating in the French West Indies

**DOI:** 10.1371/journal.pntd.0002114

**Published:** 2013-03-14

**Authors:** Pascale Bourhy, Cécile Herrmann Storck, Rafaelle Theodose, Claude Olive, Muriel Nicolas, Patrick Hochedez, Isabelle Lamaury, Farida Zinini, Sylvie Brémont, Annie Landier, Sylvie Cassadou, Jacques Rosine, Mathieu Picardeau

**Affiliations:** 1 Institut Pasteur, Unité de Biologie des Spirochètes, National Reference Center and WHO Collaborating Center for Leptospirosis, Paris, France; 2 University Hospital of Pointe à Pitre, Department of Bacteriology and Infectious and Tropical Diseases, Guadeloupe, French West Indies; 3 University Hospital of Fort de France, Department of Bacteriology and Infectious and Tropical Diseases, Martinique, French West Indies; 4 French Institute for Public Health Surveillance (InVS), Interregional epidemiology unit of French West Indies (Cire Antilles-Guyane), Fort-de-France, Martinique, French West Indies; David Geffen School of Medicine at University of California, Los Angeles, United States of America

## Abstract

**Background:**

Leptospirosis is one of the most important neglected tropical bacterial diseases in Latin America and the Caribbean. However, very little is known about the circulating etiological agents of leptospirosis in this region. In this study, we describe the serological and molecular features of leptospires isolated from 104 leptospirosis patients in Guadeloupe (n = 85) and Martinique (n = 19) and six rats captured in Guadeloupe, between 2004 and 2012.

**Methods and Findings:**

Strains were studied by serogrouping, PFGE, MLVA, and sequencing 16SrRNA and *secY*. DNA extracts from blood samples collected from 36 patients in Martinique were also used for molecular typing of leptospires via PCR. Phylogenetic analyses revealed thirteen different genotypes clustered into five main clades that corresponded to the species: *L. interrogans*, *L. kirschneri*, *L. borgpetersenii*, *L. noguchi*, and *L. santarosai*. We also identified *L. kmetyi* in at least two patients with acute leptospirosis. This is the first time, to our knowledge, that this species has been identified in humans. The most prevalent genotypes were associated with *L. interrogans* serovars Icterohaemorrhagiae and Copenhageni, *L. kirschneri* serovar Bogvere, and *L. borgpetersenii* serovar Arborea. We were unable to identify nine strains at the serovar level and comparison of genotyping results to the MLST database revealed new *secY* alleles.

**Conclusions:**

The overall serovar distribution in the French West Indies was unique compared to the neighboring islands. Typing of leptospiral isolates also suggested the existence of previously undescribed serovars.

## Author Summary

Leptospirosis is an emerging zoonotic disease caused by infection with pathogenic strains of *Leptospira*. Isolation of *Leptospira* strains is rare, making it difficult to assess their distribution worldwide. In this study, we characterized cultures of *Leptospira* obtained from more than one hundred leptospirosis patients from the French West Indies by serology and various molecular typing methods to identify the strains circulating in this endemic region. Typing of leptospiral isolates showed that causative agents of leptospirosis in the French West Indies are mainly from the serogroups Icterohaemorrhagiae and Ballum, but we also identified new genotypes. We also found that the distribution of the predominant pathogenic leptospiral serovars differed between the Caribbean islands. A better understanding of the epidemiology of leptospirosis will improve our knowledge in the distribution of this emerging neglected tropical disease worldwide. The identification of the circulating etiological agents of leptospirosis in the French West Indies will also help establish appropriate control and prevention measures in this area where the disease is endemic.

## Introduction

Leptospirosis is an emerging zoonosis with a worldwide distribution. The World Health Organization (WHO) estimates that there are over 1,700,000 severe cases of leptospirosis worldwide, with an higher incidence in impoverished populations in developing countries and tropical regions [1]–[3]. The disease is transmitted during direct contact with animal reservoirs or, more frequently, water and soil contaminated with their urine [4]. Leptospirosis is found in rural regions because of the higher risk of exposure to animal reservoirs [5], [6] and also in urban slums where inadequate sanitation provides the conditions for rat-borne transmission of the disease [7], [8]. Outbreaks may occur after heavy seasonal rainfall and extreme climatic events such as tropical storms and hurricanes [8], [9]. Leptospirosis causes a broad spectrum of symptoms from subclinical infection to multiple organ failure with a mortality rate of 10 to 50% [3].

Leptospirosis is one of the most important neglected tropical bacterial diseases in Latin America and the Caribbean [10]. This includes the French West Indies which consist of the Caribbean islands of Guadeloupe and Martinique, both French overseas departments. Climate-related changes, such as El Niño events, and periods of heavy rainfall may influence the incidence of leptospirosis in this region [11], [12]. The mean incidence of leptospirosis in the French West Indies is higher than 10/100,000 inhabitants peaking at ∼39/100,000 inhabitants in Guadeloupe in 2011, which is 100 times higher than that in Mainland France (data from the National Reference Center for Leptospirosis, Institut Pasteur).

Pathogenic *Leptospira* encompass nine species with more than 300 serovars which are the etiological agents of leptospirosis [13]. The taxonomy of the genus *Leptospira* has been both complex and controversial. Leptospiral serovars are defined by the cross agglutinin absorption test (CAAT) which uses polyclonal antibodies against the lipopolysaccharides (LPS) [4], [14]. However, this test is fastidious to perform and it is restricted to a few reference laboratories. Although the term serogroup has no taxonomic value, it has been used to define a group of antigenically related serovars which can be identified by microscopic agglutination test (MAT). Advancement in molecular techniques has allowed the speciation of members of the genus *Leptospira*. A significant outcome of the genetic classification scheme was the finding that a serovar may belong to different species [14]. With the emergence of molecular typing methods, it appears that the concept of a “serovar” is no longer fully satisfactory as it may fail to define epidemiologically important strains or genotypes.

Despite its medical significance, the isolation of clinical *Leptospira* strains is rare due to the fastidious growth in culture of this species, and poor awareness of the disease. Detailed characterization of *Leptospira* isolates is important for understanding the epidemiology of leptospirosis. *Leptospira* serovars can be prevalent in a particular geographical area and/or associated with a restricted number of animal reservoirs. Local *Leptospira* isolates can serve as antigens for the serodiagnosis of leptospirosis. The diverse distributions of *Leptospira* serovars and genotypes may have implications for vaccine design and efficacy.

The main function of the National Reference Center (NRC) for Leptospirosis, which is also a WHO Collaborating Center, at the Institut Pasteur of Paris is the surveillance of human leptospirosis. This includes the collection of diagnostic data from laboratories around the country, including French overseas territories. The NRC is the only laboratory in France that can confirm leptospirosis diagnosis by means of the MAT, which remains the gold standard for the serological diagnosis of leptospirosis, with an extended panel of antigens. The NRC also identifies clinical isolates both from mainland France and from French overseas territories. As part of a long-term typing project, more than three-quarters of all the *Leptospira* isolates received from Guadeloupe and Martinique were systematically fingerprinted so as to identify the strains circulating in this region the Americas.

## Materials and Methods

### Ethics statement

The *Leptospira* cultures from human patients analyzed in this study were previously isolated by the University Hospitals of Pointe à Pitre (Guadeloupe) and Fort de France (Martinique) and the National Reference Center for Leptospirosis (Institut Pasteur) as part of the national surveillance of leptospirosis. The strains and DNAs derived from these cultures were analyzed anonymously for this research study. All serum samples were initially sampled for diagnostic purpose, and archived at the National Reference Center for Leptospirosis (Institut Pasteur). All sera are de-linked from the patients from whom they originated and analyzed anonymously if used in any research study. The study protocol was approved by the ethical committees of the University Hospitals of Pointe à Pitre (Guadeloupe) and Fort de France (Martinique) and the CNIL (Commission Nationale Informatique et Liberté). This study was part of a protocol approved by the Institut Pasteur and the French Ministry for Education & Research French Ministry for Education & Research (protocol # AC- DC-2010-1197). Rats are listed as invasive mammals on the French West Indies [15] and authorization (through arrêté préfectoral) are regularly published, in agreement with the « Fédération Départementale des Groupements de Défense contre les Ennemis des Cultures » for their captures. Rats were captured in 2002–2003 during a study on schistosomiasis [16], which was supported by the CNRS (PNDBE), the MENRT (PRFMMIP 95) and the French Ministry of Ecology and Sustainable Development (Contract CV 02000071, MEDD, programme “Ecosystemes Tropicaux”). Capture and euthanasia of rats was performed by Dr. A. Theron (University of Perpignan) who was accredited to experiment on rodent (authorization n° C 66.11.02 by the Préfecture des Pyrénées Orientales). Studies on rats were performed in accordance with the European Union legislation (Directive 86/609/EEC).

### Diagnosis of leptospirosis by MAT and PCR

Serum samples from suspected cases of leptospirosis were subjected to the microscopic agglutination test (MAT) at the National Reference Center for Leptospirosis (NRC) at the Institut Pasteur (Paris, France). MAT was performed using 24 leptospiral antigens (**Table S1**). A high agglutination titer of the serum with one particular serogroup is taken to identify the presumptive serogroup of the infecting bacterium. For patients presenting symptoms during the first week of infection, total genomic DNA was extracted from plasma collected into EDTA tubes and tested for the presence of pathogenic *Leptospira* by real-time PCR [17]. Molecular Biology Grade Water (EUROBIO, Les Ulis, France) was used for PCR. Reactions with no template DNA were included as negative controls in each PCR experiment. For patients testing positive by PCR, acute and, if possible, convalescent serum samples were collected for serological testing.

Leptospirosis cases were defined as having clinical signs and symptoms consistent with leptospirosis and a single MAT titer ≥1/400 for a pathogenic serogroup or detection of pathogenic *Leptospira* by PCR or culture.

### Leptospiral strain and DNA isolation from blood

A total of 104 clinical isolates of *Leptospira* isolated from patients in Guadeloupe (85) and Martinique (19) between 2004 and 2012 were studied. *Leptospira* was cultured by inoculating plasma prepared from heparinized blood from patients into EMJH liquid medium [18] at the University Hospitals of Pointe à Pitre (Guadeloupe) and Fort de France (Martinique). Leptospires positive cultures were then sent to the NRC for Leptospirosis (Institut Pasteur, Paris, France) for typing. Six isolates collected from kidney tissues of *Rattus rattus* captured in Guadeloupe (mangrove area of Morne à L'eau) in 2002–2003 (André Théron, University of Perpignan) were also included in the study. Reference strains from the collection maintained by the NRC for Leptospirosis were used for comparisons (**Table S2**). DNA was also isolated from the blood of 36 additional patients from Martinique who tested positive for leptospirosis by PCR during the study.

### Serological characterization of isolates

The microscopic agglutination test (MAT) was used for antigenic characterization of *Leptospira* isolates, with a standard battery of rabbit antisera against reference serovars representing the 24 serogroups as previously described [18]. Mice monoclonal antibodies F70 C14-10 and F70 C24-20 (WHO/FAO/OIE and National Collaborating Centre for Reference and Research on Leptospirosis, Royal Tropical Institute, Amsterdam, The Netherlands), which react against the serovars Icterohaemorrhagiae and Copenhageni respectively, were also used for some strains as previously described [19].

### Genetic characterization of *Leptospira*

Genomic DNA was extracted from EMJH cultures or from human plasma (see above). DNA was amplified using Taq polymerase (GE Healthcare) under standard conditions. For species identification, the *rrs* gene was amplified with the primers LA (5′-GGCGGCGCGTCTTAAACATG-3′) and LB (5′-TTCCCCCCATTGAGCAAGATT-3′), and when necessary, by nested primers LC (5′-CAAGTCAAGCGGAGTAGCAA-3′) and RS4 (5′- TCTTAACTGCTGCCTCCCGT-3′) [20], [21]. Part of the *secY* gene was amplified with the primers F (5′-ATGCCGATCATTTTTGCTTC-3′) and R (5′-CCGTCCCTTAATTTTAGACTTCTTC-3′) [22]. Sequencing was performed at the Genotyping of Pathogens and Public Health Platform (Institut Pasteur, Paris, France). All molecular epidemiological data were stored and analysed with Bionumerics software (Version 6.5; Applied-Maths, Belgium). Genotyping was also performed by multiple-locus variable-number tandem repeat analysis (MLVA) using the loci VNTR4, VNTR7, and VNTR10 as described by Salaun *et al.* [23]. In the absence of PCR products, a second round of nested PCR amplification was performed with the inner primers NP 4A (5′-TTGGAGCGCAATCTCTTTTT-3′) and NP4B (5′- TGAGGATACCCCATTTTTACCTT-3′), NP7A (5′-GATGGGCGGAGAAAAGTGTA-3′) and NP7B (5′-TGGATCGGTATTTTGGTTCA- 3′), NP10A (5′-ATTCCAAAACTCAGCCCTCA-3′) and NP10B (5′- TGATGGGATTACCGGAAGAA-3′). For pulsed-field gel electrophoresis (PFGE), cells were embedded in agarose plugs as previously described [24], and the DNA in the plugs digested with *Not*I. PFGE was performed in a contour-clamped homogeneous electric field DRII apparatus (Bio-Rad Laboratories, Richmond, CA). Restriction fragments were resolved with ramping from 5 to 60 s for 50 h, 1 to 30 s for 40 h, or from 1 to 70 s for 36 h at 6 V/cm.

### Nucleotide sequence accession numbers

Nucleotide sequences have been deposited with GenBank under accession numbers JX827500 - JX827597.

## Results

### Diagnosis of leptospirosis in the French West Indies from 2007 to 2011

Guadeloupe and Martinique are islands situated in the Caribbean archipelago and are 100 miles apart. Guadeloupe and Martinique share common geological environments (although Grande Terre in Guadeloupe is composed of limestone, the islands are mainly volcanic) and are 1,705 and 1,100 km^2^, respectively. They have similar population sizes (<400,000 inhabitants) and levels of urbanization. The islands are among the most highly developed islands in the Caribbean and their economies depend largely on tourism and agriculture (sugar cane and bananas). The climate is tropical with two distinct seasons, the dry season from December to May and the rainy season from June to November.

Over the last five years (2007–2011), the annual incidence of leptospirosis has ranged from <12 per 100,000 inhabitants in Martinique (2007) to >41 per 100,000 inhabitants in Guadeloupe (2011) (data from the NRC for Leptospirosis, Institut Pasteur, France), which is among the highest reported in the Caribbean (<2 per 100,000 inhabitants in Trinidad and Tobago [25], [26] and <13/100,000 inhabitants in Barbados [27]).

In 2011, the total number of cases was 165 in Guadeloupe and 142 in Martinique, which is two to three-fold more than in 2007. Most of the infections were during the rainy season from August to November (around 70% of all cases in 2011).

Detection of antibodies in patient sera by MAT has shown that the most prevalent *Leptospira* serogroup in the French West Indies is Icterohaemorrhagiae (<25% in Martinique and <37% in Guadeloupe). The other serogroups each account for less than 12% of cases and include serogroups Ballum (<5% in Martinique and <12% in Guadeloupe), Sejroe (<7% in Martinique and <5% in Guadeloupe), and Canicola (<7% in Martinique and <9% in Guadeloupe) (data from the NRC for Leptospirosis, Institut Pasteur, France).

### Identification of circulating pathogenic *Leptospira*

Results of identification of strains sent to the NRC for Leptospirosis (Institut Pasteur) for serogroup and genotype identification are shown in Table 1 (**Table 1**). Genomic DNA from 36 acute-phase blood samples that were positive for pathogenic *Leptospira* by PCR at the University Hospital of Fort de France were also included in this study (**Table 2**). The geographical distribution of the isolates was as follows: 91 strains isolated in Guadeloupe from 2003 to 2012 (including 6 rat isolates) and 55 strains isolated in Martinique from 2011 to 2012 (including DNA from 36 patients) (**Tables 1** **and** **2**).

**Table 1:** Identification of isolates sent to the NRC for Leptospirosis.

Origin	Source (number)	Year of isolation	Species (*rrs*)	Serogrouping (MAT)	Presumptive serovar (PFGE)	MLVA^b^	*secY*
Guadeloupe	rat (1)	2002	*L. interrogans*	Ictero	Ictero/Copenhageni	500/350/750	A
	human (4)	2007	*L. interrogans*	Ictero	Ictero/Copenhageni	500/350/750	A
	human (3)	2008	*L. interrogans*	Ictero	Ictero/Copenhageni	500/350/750	A
	human (2)	2009	*L. interrogans*	Ictero	Ictero/Copenhageni	500/350/750	A
	human (3)	2010	*L. interrogans*	Ictero	Ictero/Copenhageni	500/350/750	A
	human (5)	2011	*L. interrogans*	Ictero	Ictero/Copenhageni	500/350/750	A
	human (7)	2012	*L. interrogans*	Ictero	Ictero/Copenhageni	500/350/750	A
Martinique	human (2)	2011	*L. interrogans*	Ictero	Ictero/Copenhageni	500/350/750	A
	human (4)	2012	*L. interrogans*	Ictero	Ictero/Copenhageni	500/350/750	A
Guadeloupe	rat (4)	2003	*L. kirschneri*	Ictero	Bogvere	380/560/880	B
	human (3)	2004	*L. kirschneri*	Ictero	Bogvere	380/560/880	B
	human (5)	2005	*L. kirschneri*	Ictero	Bogvere	380/560/880	B
	human (3)	2006	*L. kirschneri*	Ictero	Bogvere	380/560/880	B
	human (2)	2007	*L. kirschneri*	Ictero	Bogvere	380/560/880	B
	human (4)	2010	*L. kirschneri*	Ictero	Bogvere	380/560/880	B
	human (5)	2011	*L. kirschneri*	Ictero	Bogvere	380/560/880	B
	human (5)	2012	*L. kirschneri*	Ictero	Bogvere	380/560/880	B
Martinique	human (1)	2011	*L. kirschneri*	Ictero	Bogvere	380/560/880	B
	human (1)	2012	*L. kirschneri*	Ictero	Bogvere	380/560/880	B
Guadeloupe	rat (1)	2002	*L. borgpetersenii*	Ballum	Castellonis/Arborea	ND	C
	human (2)	2004	*L. borgpetersenii*	Ballum	Castellonis/Arborea	ND	C
	human (6)	2007	*L. borgpetersenii*	Ballum	Castellonis/Arborea	ND	C
	human (1)	2008	*L. borgpetersenii*	Ballum	Castellonis/Arborea	ND	C
	human (2)	2010	*L. borgpetersenii*	Ballum	Castellonis/Arborea	ND	C
	human (11)	2011	*L. borgpetersenii*	Ballum	Castellonis/Arborea	ND	C
	human (2)	2012	*L. borgpetersenii*	Ballum	Castellonis/Arborea	ND	C
Martinique	human (1)	2011	*L. borgpetersenii*	Ballum	Castellonis/Arborea	ND	C
	human (1)	2012	*L. borgpetersenii*	Ballum	Castellonis/Arborea	ND	C
	human (3)	2012	*L. borgpetersenii*	Tarassovi	unknown	ND	D
Guadeloupe	human (1)	2011	*L. noguchi*	Australis	Bajan	ND	E
Guadeloupe	human (1)	2004	*L. santarosai*	Mini	Tabaquite	ND	G
	human (1)	2007	*L. santarosai*	Mini	Tabaquite	ND	G
	human (1)	2010	*L. santarosai*	Mini	Tabaquite	ND	G
	human (5)	2011	*L. santarosai*	Mini	Tabaquite	ND	G
	human (1)	2012	*L. santarosai*	Mini	Tabaquite	ND	G
Martinique	human (2)	2012	*L. santarosai*	unknown^a^	unknown	ND	H
	human (1)	2011	*L. santarosai*	Celledoni	unknown	ND	I
	human (1)	2011	*L. santarosai*	unknown^a^	unknown	ND	J
	human (1)	2012	*L. santarosai*	unknown^a^	unknown	ND	J
	human (1)	2011	*L. santarosai*	Tarrasovi^a^	unknown	ND	L

ND: not determined.

Ictero: Icterohaemorragiae.

a : no agglutination with rabbit antisera against reference strains representative of 24 serogroups.

b : size of the PCR products (in bp) for VNTR4, VNTR7, and VNTR10.

**Table 2:** Identification of Leptospira DNA from acute-blood samples.

DNA	Species (*rrs*)	MLVA^c^	*secY*	MAT titer with serum sample (s)	Presumptive serogroup/serovar^d^
201100327	*L. interrogans*	ND	A	NA	Ictero/Ictero
201101569	*L. interrogans*	500/350/750	A	Ictero 1,600	Ictero/Ictero
201102939	*L. interrogans*	500/350/750	A	Ictero 1,600	Ictero/Ictero
201102942	*L. interrogans*	500/350/750	A	negative	Ictero/Ictero
201102943	*L. interrogans*	500/350/750	A	negative	Ictero/Ictero
201102949	*L. interrogans*	500/350/ND	A	negative	Ictero/Ictero
201103357	*L. interrogans*	500/350/750	A	Ictero 1,600	Ictero/Ictero
201103358	*L. interrogans*	500/350/750	A	NA	Ictero/Ictero
201103359	*L. interrogans*	500/350/750	A	negative	Ictero/Ictero
201203644	*L. interrogans*	500/350/750	A	Patoc 12,800	Ictero/Ictero
201203646	*L. interrogans*	500/350/750	A	Ictero 800	Ictero/Ictero
201102941	*L. interrogans*	ND	K	NA	unknown/unknown
201102942	*L. interrogans*	ND	K	NA	unknown/unknown
201103361	*L. kirschneri*	380/560/ND	B	Ictero + Cynopteri 1,600	Ictero/Bogvere
201202631	*L. kirschneri*	380/560/ND	B	NA	Ictero/Bogvere
201203648	*L. kirschneri*	ND	ND	Patoc 100	unknown/unknown
201101574	*L. borgpetersenii*	ND	C	NA	Ballum/Arborea
201102940	*L. borgpetersenii*	ND	C	Ictero 3,200	Ballum/Arborea
201102947	*L. borgpetersenii*	ND	C	Ballum 1,600	Ballum/Arborea
201103360	*L. borgpetersenii*	ND	C	negative	Ballum/Arborea
201201366	*L. borgpetersenii*	ND	C	NA	Ballum/Arborea
201202629	*L. borgpetersenii*	ND	C	NA	Ballum/Arborea
201200550	*L. borgpetersenii*	ND	D	Patoc 200	Tarassovi/unknown
201103366	*L. borgpetersenii*	ND	D	NA	Tarassovi/unknown
201103369	*L. noguchi*	ND	E	Australis 200	Australis/Bajan
201201371	*L. noguchi*	ND	E	NA	Australis/Bajan
201102111	*L. kmetyi* a	ND	ND	Patoc 800	unknown/unknown
201103362	*L. kmetyi* a	ND	ND	Patoc 400	unknown/unknown
201102109	*L.* kmetyi/*L.kirschneri* b	ND	M	NA	unknown/unknown
201103368	*L. santarosai*	ND	H	Patoc 400	unknown/unknown
201103365	*L. santarosai*	ND	I	Celledoni 3,200	Celledoni/unknown
201200547	*L. santarosai*	ND	I	NA	Celledoni/unknown
201200548	*L. santarosai*	ND	I	Patoc 400	Celledoni/unknown
201202627	*L. santarosai*	ND	I	NA	Celledoni/unknown
201203642	*L. santarosai*	ND	I	Patoc 800 Ballum 800	Celledoni/unknown
201201370	*L. santarosai*	ND	J	NA	unknown/unknown

NA: non applicable.

Ictero: Icterohaemorragiae.

a : Partial sequencing of the 5′ variable region of the 16S rRNA gene of 201102111 and 201103362 showed 99% (277/279 nucleotides) with the reference strain of *L. kmetyi*.

b : Partial sequencing of the 5′ variable region of the 16S rRNA gene of 201102109 showed identities to both *L. kmetyi* (273/279 nucleotides) and *L. kirschneri* (272/279 nucleotides).

c : size in bp of the PCR products for VNTR4, VNTR7, and VNTR10.

d : serovars Icterohaemorragiae and Copenhageni cannot be distinguished by molecular methods.

Serogrouping of isolates was first performed with rabbit antisera against reference serovars. The most frequent serogroups were Icterohaemorrhagiae (58%) and Ballum (25%), consistent with the findings obtained by MAT with human serum samples (see above). Other serogroups detected include Mini (9 isolates), Tarassovi (4 isolates), Australis (1 isolate), and Celledoni (1 isolate). Four isolates scored negative (no agglutination) with the antisera raised against the 24 serogroups. A selection of isolates from the serogroup Icterohaemorrhagiae were subsequently typed to serovar level by MAT with monoclonal antibodies (MAbs) against the serovars Icterohaemorrhagiae and Copenhageni: both serovars were present among the clinical isolates (data not shown).

**Figure 1:**
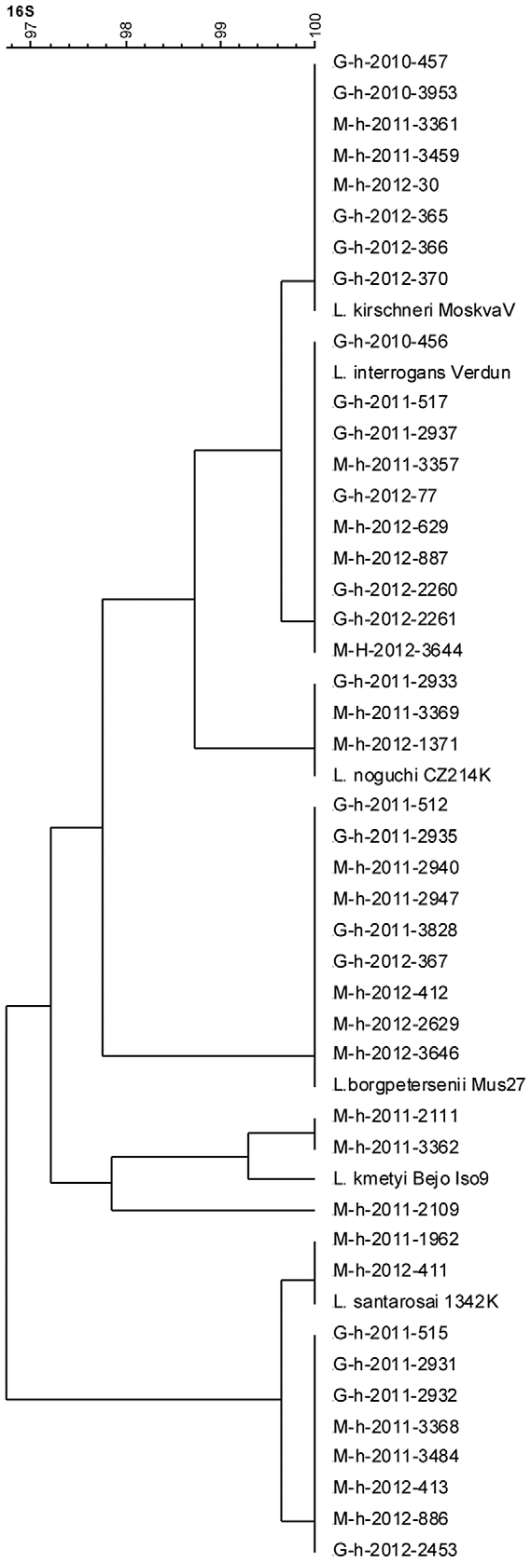
Phylogenetic tree of leptospiral 16S rRNA gene sequences of reference strains (*L. kirschneri* serovar Grippotyphosa strain MoskvaV, *L. interrogans* serovar Icterohaemorrhagiae strain Verdun, *L. santarosai* serovar Shermani strain 1342K, *L. borgpetersenii* serovar Ballum strain Mus27, and *L. noguchi* serovar Panama strain CZ214K) and a set of clinical isolates from Guadeloupe (G) and Martinique (M). The tree was drawn using the UPGMA (unweighted pair group method with arithmetic average) algorithm.

Molecular typing was then performed by sequencing the 16S rRNA gene (*rrs*) in genomic DNA from 110 cultures and 36 acute-blood samples [20]. All the samples corresponded to one of five pathogenic species: *L. interrogans* (44 samples), *L. kirschneri* (36 samples), *L. borgpetersenii* (38 samples), *L. noguchi* (3 samples), and *L. santarosai* (22 samples) (**Tables 1** **and** **2**). Two samples were phylogenetically related to *L. kmetyi* (**Figure 1**). The sequences of their 279-nucleotide 16S rRNA PCR products were identical with two mismatches (99% nucleotide identity) to the corresponding variable region of the 16S rRNA sequence of the *L. kmetyi* reference strain. These *L. kmetyi*-positive cases showed MAT cross reaction with the saprophyte serovar Patoc (**Table 2**). The serovar Patoc, which is non-pathogenic, was included in our analysis because it has cross-reactivity with pathogenic serogroups and can be indicative of an infection. The last sample (201102109) was related to both *L. kmetyi* (273/279 nucleotides) and *L. kirschneri* (272/279 nucleotides). This DNA may therefore correspond to a variant of *L. kmetyi* or *L. kirschneri*.

**Figure 2:**
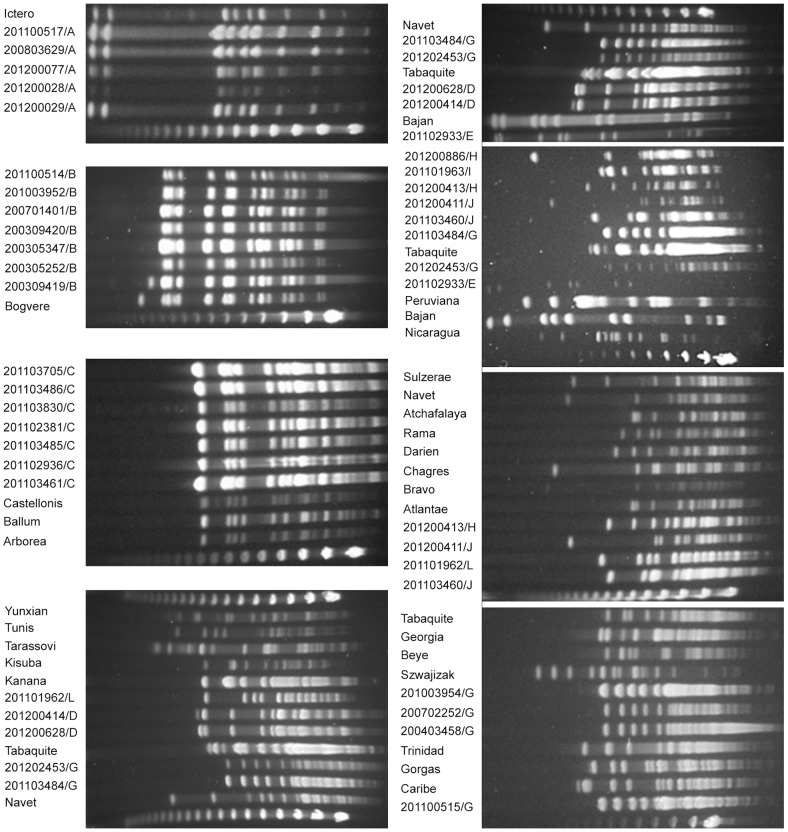
Representative PFGE patterns of NotI-digested genomic DNA from isolates from Martinique and Guadeloupe. The genotype is indicated for each clinical isolate, and reference strains for serovars Panama, Icterohaemorragiae, Tbaquite, Bogvere, Beye, Szwajizak, Trinidad, Gorgas, Caribe, Ballum, Castellonis, Arborea, Sulzeae, Navet, Atchafalaya, Rama, Darien, Chagres, Bravo, Nicaragua, Bajan, Peruviana, and Atlantae. The molecular weight size marker is bacteriophage lambda DNA concatemers of 50 kb.

PFGE has long been the gold standard method for genotyping *Leptospira* strains [28], [29]. PFGE analysis of *Not*I-digested genomic DNA revealed at least thirteen distinct patterns for the typed isolates (**Figure 2**). For each strain, serovar designation was attributed by comparing the patterns with those of reference strains belonging to the identified serogroup and species (**Table S2**). For example, patterns of isolates identified as belonging to the species *L. santarosai* and serogroup Mini were compared with reference serovars that belong to the *L. santarosai* serogroup Mini (i.e. serovars Beye, Georgia, Szwajizak, and Tabaquite). In this case, the “Mini” isolates displayed a PFGE pattern which was similar (less than three band differences) to the type strain of serovar Tabaquite (**Figure 2**). The “Tarassovi” isolates displayed unique PFGE patterns which were different from the PFGE patterns of the reference strains of *L. borgpetersenii* and *L. santarosai* serogroup Tarassovi (serovars Kisuba, Tarassovi, Kanana, Guidae, Tunis, Yunxian, Atchafalaya, Atlantae, Bravo, Chagres, Darien, Navet, Rama, and Sulzerae). The PFGE profile of the “Australis” isolate was similar to serovar Bajan and distinct to the other reference strains from *L. noguchi* serogroup Australis (serovars Rushan, Peruviana, and Nicaragua). For the “Celledoni” isolate, none of the reference serovars within this serogroup belong to the species *L. santarosai.* The PFGE patterns of the “Icterohaemorrhagiae” strains, which were subdivided into *L. interrogans* or *L. kirschneri*, were identical to the patterns obtained from *L. interrogans* serovars Icterohaemorrhagiae and Copenhageni, known to be indistinguishable by PFGE and other molecular typing techniques, and *L. kirschneri* serovar Bogvere (less than three band differences were observed). The “Icterohaemorrhagiae” strains that were isolated from different patients over an eight-year period (2004–2012) and those from rats all presented indistinguishable PFGE patterns. The “Ballum” isolates displayed a pattern that was similar to that displayed by *L. borgpetersenii* serovars Ballum, Castellonis, Guangdong, Arborea, and Soccoestomes [30] (**Figure 2**).

MLVA (Multi Locus VNTR Analysis) is a simple and rapid PCR-based method for the identification of most of the serovars of *L. interrogans* and *L. kirschneri* [23]. The *L. interrogans* and *L. kirschneri* isolates from the French West Indies had a MLVA pattern with VNTR-4, VNTR-7, and VNTR-10 identical to the serovars Icterohaemorrhagiae and Bogvere type strains, respectively. This is in agreement with the clusters determined by PFGE (**Table 1**), further confirming the identity of the serovars Icterohaemorrhagiae/Copenhageni and Bogvere. Strains from species *L. borgpetersenii*, *L. noguchi*, *L. santarosai*, and *L. kmetyi* could not be typed by this method because of the absence of one or more of the VNTR loci.

**Figure 3:**
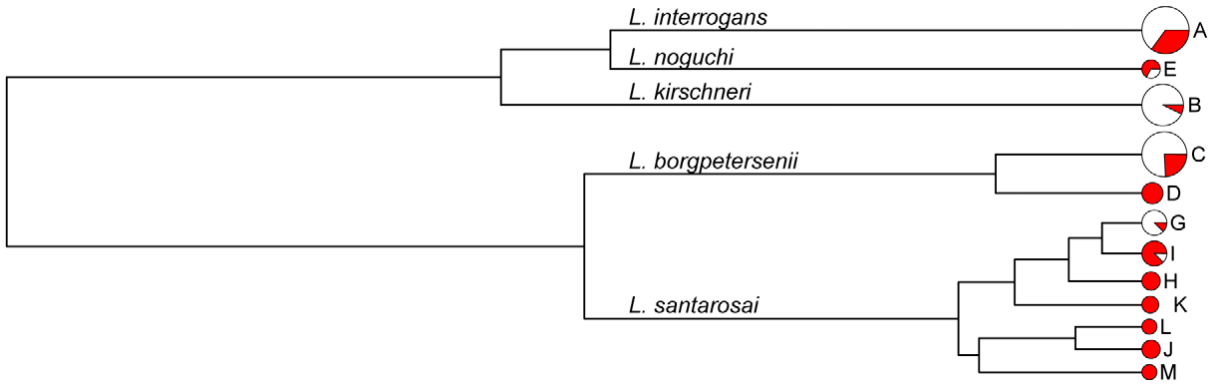
Phylogenetic relationships of leptospirosis isolates based on *secY* sequences. The tree was drawn using the UPGMA (unweighted pair group method with arithmetic average) algorithm. The species and genotype are indicated. Circle sizes correspond to the numbers of strains of each genotype. Isolates from Martinique are highlighted by a red background.

The *secY* housekeeping gene [22] was also amplified from DNA extracts and sequenced. No PCR products were obtained for DNA from the *L. kmetyi* strains (here designated as genotype F). This was presumably due to mismatching between the PCR primers and the target gene (due to DNA sequence divergence), preventing PCR amplification [31]. The phylogenetic tree constructed with the *secY* nucleotide sequences is shown in the Figure 3 (**Figure 3**). Our 143 sequences (not including the 3 *L. kmetyi* strains) segregate into five main clades that correspond to the species identified by 16S rRNA sequencing. Thirteen different genotypes were observed and genotypes A (42 isolates), B (35 isolates), and C (33 isolates) were the most prevalent. The *secY* alleles A, B, and C were associated with serovars Icterohaemorrhagiae/Copenhageni, Bogvere, and Arborea/Castellonis/Ballum/Guangdong/Soccoestomes, respectively. The remaining 32 strains were distributed into nine groups (D, E, G, H, I, J, K, L, and M), including six new alleles not found in the database published by Nalam *et al.* [32]. Thus, there were thirteen groups in total, and most were present on both Guadeloupe and Martinique. However, some genotypes were found only among isolates from Guadeloupe (group G with 9 isolates) or Martinique (groups H with 3 samples, I with 6 samples, and J with 2 isolates). Clusters A and B contained both, clinical and rat isolates.

## Discussion

Leptospirosis is endemic in the French West Indies. The first human cases were first documented in 1932 in Guadeloupe [33] and 1938 in Martinique [34]. The annual incidence of leptospirosis in the French West Indies was estimated to be approximately 10 cases per 100,000 inhabitants in the 1990s. The incidence of leptospirosis during 2002–2004 was affected by the El Nino phenomenon, which resulted in increases in rainfall and the number of cases in Guadeloupe [35]. A prospective study of patients with acute febrile illness in Martinique and Guadeloupe (InVS, CIRE Antilles-Guyane) in 2011 improved the surveillance of leptospirosis. This increased awareness could explain the record incidence in 2011, peaking at <39 cases per 100,000 inhabitants in both Guadeloupe and Martinique.

Although the use of PCR diagnostic testing is becoming more common in the French West Indies, the diagnosis of leptospirosis is mostly dependent on MAT, which can identify the presumptive serogroup of the infecting bacterium. MAT has been used to show that the most frequent serogroups in Guadeloupe are Icterohaemorrhagiae and Ballum, followed by Sejroe and Canicola [36] (data from the NRC for Leptospirosis). Serogroups Cynopteri, Tarassovi, Panama, Grippotyphosa and Autumnalis are less common. The sensitivity of MAT is low during the acute stage of disease [37] and, because of paradoxical reactions and cross-reactions between serogroups, the accuracy of MAT in identifying the infecting serovar or serogroup can also be poor [38], [39], limiting its epidemiological value. In this study, MAT serological data from culture-positive patients were reviewed retrospectively, allowing the identification of a total of 36 patients with MAT data for serum samples (data not shown). It was possible to infer the serogroup identity of infecting leptospires from the MAT results for 26 of these 36 patients (72%). Similarly, only a small proportion of PCR-positive samples were correctly identified by MAT (Table 2). This further confirms that only the isolation of *Leptospira* from patients allowed definitive identification of the infecting serovar and is therefore essential for the study of the epidemiology of the disease.

We determined 16S rRNA sequences to identify the isolates to the species level, and then used serogrouping, PFGE, *secY* sequences, and MLVA to sub-type the species.

For most of the isolates (101/110), the PFGE patterns were mostly consistent with those of known serovars: i.e. serovars Bogvere, Tabaquite, Bajan, and Icterohaemorrhagiae or Copenhageni. For the serogroup Ballum, the PFGE patterns of the reference isolates for serovars Ballum, Castellonis, Guangdong, Arborea, and Soccoestomes were all similar, with fewer than three band differences [30]. Serovar Arborea was previously identified by CAAT as the major serovar from the serogroup Ballum in the Caribbean island Barbados [40], suggesting that our strains may belong to serovar Arborea. For the remaining five isolates which were serogrouped (Tarassovi and Celledoni), comparison of PFGE patterns with reference strains was inconclusive for the serovar. Finally, for four isolates the rabbit antisera used did not lead to agglutination such that comparison with the reference strains was not possible. Surprisingly, none of the isolates in the last ten years from the French West Indies were identified as belonging to serogroups Canicola or Sejroe, although up to14% of MAT-positive sera correspond to these two serogroups. Similar findings were reported in Barbados [40]. This may be due to cross-reactions between serogroups in MAT and/or difficulties in isolating these strains from patients (for example patients not hospitalized because of less severe symptoms or the strains fail to grown in EMJH medium).

Typing by PCR-based methods for amplification of 16S rRNA, *secY*, and VNTR loci can be used directly on biological samples, thus avoiding culturing of the pathogen. The bacterial load in blood during the acute phase ranges from 10^2^ to 10^6^ *Leptospira*/ml. The *Leptospira* count decreases with time, and can be detected for up to 15 days [41]. Thus, if the bacterial load is low, it may be necessary to use nested-PCR for amplification of the target sequences. The classification according to *secY* sequences was in good agreement with the groupings determined by PFGE and MLVA, further confirming our previous data on clinical isolates from Mayotte [42].

Sequencing of *secY* in DNA extracted from the clinical isolates and blood samples allowed a simple and rapid first-line screening and the identification of the presumptive serovar. A total of thirteen genotypes were found in our study, a large proportion of strains (75%) being of only three genotypes associated with serovars Icterohaemorrhagiae/Copenhageni, Bogvere, and Arborea. The *secY* sequences from the *L. santarosai* isolates showed the highest nucleotide diversity. Six genotypes were not found in the MLST database and may therefore be specific to the French West Indies. Further characterization of these isolates should include the use of the CAAT, which requires the preparation of antibodies against the strain of interest, for definitive identification of the serovar. We also detected the appearance of strains related to the pathogenic species *L. kmetyi* in Martinique in at least two patients, one of which was probably exposed during canyoneering activities in the tropical forest (Hochedez *et al.*, submitted). To our knowledge, *L. kmetyi* which was first isolated from soil in Malaysia [43], has never been isolated from a patient with leptospirosis. Further studies are needed to determine the serological and molecular features of these strains and their distribution in the French West Indies.

The distribution of the predominant pathogenic leptospiral serovars differed between Guadeloupe and Martinique. Serovars Bogvere, Arborea, and Icterohaemorrhagiae/Copenhageni made up 35, 31, and 23% respectively of all *Leptospira* isolates in Guadeloupe since 2004. In Martinique, serovar Icterohaemorrhagiae is the most frequent (35%), followed by Arborea (9%) and Bogvere (6%). In the Caribbean island of Barbados, 140 miles from Martinique, serovars Arborea (14%) and Icterohaemorrhagiae (26%) similarly cause many human infections, but the serovar Bogvere [38], [40], [44], which was first isolated in Jamaica [45] does not. Serovar Tabaquite (serogroup Mini), which was found in Guadeloupe, was first isolated from a patient in Trinidad [46]. Serovar Bim (serogroup Autumnalis) is the most frequently isolated serovar in Barbados (75% of all isolates) [40], was not isolated in the French West Indies. This suggests that some strains circulate throughout the Caribbean islands but others are highly prevalent only in restricted areas. This may be related to the distribution of the animal reservoirs for the different serovar in these islands.

*Leptospira* can colonize or infect renal tubules of a wide variety of wild and domesticated mammals. In the Caribbean, numerous mammalian species including rodents, opossums, mongoose, bats, pigs, cattle, and dogs have been demonstrated to be hosts of pathogenic *Leptospira* species [47]. Isolates from the serogroup Icterohaemorrhagiae, including serovars Icterohaemorrhagiae and Bogvere, have been isolated from the kidneys of rats, mice, and mongoose and isolates from the serogroup Ballum were isolated from rats and mice, suggesting predominantly rodent-borne transmission of the disease. Serovar Arborea was reported to be prevalent in both humans and animals in Barbados [40], [48]. Serovar Bajan was originally isolated from toads and frogs in Barbados [49]. In our study, human and rat isolates from Guadeloupe and belonging to serovars Icterohaemorrhagiae/Copenhageni, Bogvere, and Arborea all showed identical genotypes, consistent with rats being responsible for the transmission of the disease.

The identification of the circulating etiological agents of leptospirosis in the French West Indies will help establish appropriate control and prevention measures in this area where the disease is endemic. For example, the reference technique, MAT, requires a panel of live antigens representing a broad range of serogroups. The use of local isolates in the panel of antigens may maximize the chances of detecting an immune response to the infecting bacterium. At the NRC for Leptospirosis (Institut Pasteur), the initial panel of 18 antigens, which already included strains representative of the serogroups Icterohaemorrhagiae, Ballum, Australis and Tarassovi, was thus expanded to include local isolates from serogroups Celledoni and Mini for serum samples originating from the French West Indies. Knowledge of leptospiral epidemiology may also be useful for the development of a whole bacterial vaccine against leptospirosis. Vaccines currently available for use in animals and, in a few countries, in humans generally consist of one, two or more locally prevalent serovars. In France, a human vaccine containing only serovar Icterohaemorrhagiae has been used since 1981 [50]. However, we report here that only one-third of the infections in the French West Indies are due to serovar Icterohaemorrhagiae (not including serovar Bogvere from the serogroup Icterohaemorrhagiae), and the corresponding figure for Barbabos is 22.5% [38]. Immunity is restricted to antigenically related serovars, so the vaccine used in France may not be effective against the majority of strains circulating in the French West Indies.

Further studies should include the analysis of the influence of serovar and strain genetic background on the clinical presentation and outcome of the disease. It would also be valuable to investigate the reasons for differences in the distributions of *Leptospira* serovars in the Caribbean islands.

## Supporting Information

Table S1 List of leptospiral antigens for MAT.
(DOCX)

Table S2 Reference strains used in this study.
(DOCX)
