# Supplementary material for: Serovar Diversity of Pathogenic Leptospira Circulating in the French West Indies
Source: PLoS Negl Trop Dis. 2013 Mar 14;7(3):e2114. doi: 10.1371/journal.pntd.0002114 (PMC3597474; doi:10.1371/journal.pntd.0002114)
Supplement: Table S1 — List of leptospiral antigens for MAT. (DOCX) [file pntd.0002114.s001.docx]

| **Table S1: List of leptospiral antigen used for MAT** | | |  |
| --- | --- | --- | --- |
|  |  |  |  |
| *Species* | Serogroup | Serovar | Strain |
|  |  |  |  |
| *L. interrogans* | Australis | Australis | Ballico |
| *L. interrogans* | Autumnalis | Autumnalis | Akiyami A |
| *L. interrogans* | Bataviae | Bataviae | Van Tienen |
| *L. interrogans* | Canicola | Canicola | Hond Utrecht IV |
| *L. borgpetersenii* | Ballum | Castellonis | Castellon 3 |
| *L. kirschneri* | Cynopteri | Cynopteri | 3522 C |
| *L. kirschneri* | Grippotyphosa | Grippotyphosa | Moskva V |
| *L. interrogans* | Sejroe | Hardjobovis | Sponselee |
| *L. interrogans* | Hebdomadis | Hebdomadis | Hebdomadis |
| *L. interrogans* | Icterohaemorrhagiae | Copenhageni | Wijnberg |
| *L. noguchii* | Panama | Panama | CZ 214 K |
| *L. biflexa* | Semaranga | Patoc | Patoc 1 |
| *L. interrogans* | Pomona | Pomona | Pomona |
| *L. interrogans* | Pyrogenes | Pyrogenes | Salinem |
| *L. borgpetersenii* | Sejroë | Sejroë | M 84 |
| *L. borgpetersenii* | Tarassovi | Tarassovi | Mitis Johnson |
| *L. interrogans* | Icterohaemorrhagiae | Icterohaemorrhagiae | Verdun |
| *L. weilii* | Celledoni | ND | 2011/01963 |
| *L. interrogans* | Djasiman | Djasiman | Djasiman |
| *L. borgpetersenii* | Mini | ND | 2008/01925 |
| *L. weilii* | Sarmin | Sarmin | Sarmin |
| *L. santarosai* | Shermani | Shermani | 1342 K |
| *L. borgpetersenii* | Javanica | Javanica | Poi |
| *L. noguchii* | Louisiana | Louisiana | LUC1945 |
|  |  |  |  |
| ND: Non determined | |  |  |
