# Supplementary material for: Serovar Diversity of Pathogenic Leptospira Circulating in the French West Indies
Source: PLoS Negl Trop Dis. 2013 Mar 14;7(3):e2114. doi: 10.1371/journal.pntd.0002114 (PMC3597474; doi:10.1371/journal.pntd.0002114)
Supplement: Table S2 — Reference strains used in this study. (DOCX) [file pntd.0002114.s002.docx]

| **Table S2: Reference strains used in this study** | | | |  |  |
| --- | --- | --- | --- | --- | --- |
|  |  |  |  |  |  |
| **Species** | **Serogroup** | **Serovar** | **Strain** | **Country** | **Source** |
| *L. borgpetersenii* | Celledoni | Anhoa | LT 90-68 | Vietnam | Human |
| *L. borgpetersenii* | Celledoni | Whitcombi | Whitcomb | Malaysia | Human |
| *L. borgpetersenii* | Ballum | Arborea | Arborea | Italy | Wood Mouse |
| *L. borgpetersenii* | Ballum | Castellonis | Castellon 3 | Spain | Wood Mouse |
| *L. borgpetersenii* | Ballum | Ballum | Mus 127 | Danmark | Field mouse |
| *L. borgpetersenii* | Tarassovi | Guidae | RP 29 | Brazil | Pig |
| *L. borgpetersenii* | Tarassovi | Kanana | Kanana | Kenya | Gerbil |
| *L. borgpetersenii* | Tarassovi | Kisuba | Kisuba | Zaire | Human |
| *L. borgpetersenii* | Tarassovi | Tarassovi | Perepelitsin | Russia | Human |
| *L. borgpetersenii* | Tarassovi | Tunis | P 2/65 | Tunisia | Pig |
| *L. borgpetersenii* | Tarassovi | Yunxian | L 100 | China | Pig |
| *L. interrogans* | Icterohaemorrhagiae | Copenhageni | Wijnberg | Holland | Human |
| *L. interrogans* | Icterohaemorrhagiae | Icterohaemorrhagiae | RGA | Belgium | Human |
| *L. kirschneri* | Icterohaemorrhagiae | Bogvere | LT 60-69 | Jamaica | Rat |
| *L. kmetyi* | Tarassovi | unknown | Bejo-Iso9(T) | Malaysia | soil |
| *L. noguchi* | Panama | Panama | CZ 214 K | Panama | Opossum |
| *L. noguchi* | Australis | Rushan | 507 | China | *Bombina orientalis* |
| *L. noguchi* | Australis | Nicaragua | 1011 | Nicaragua | Mustela nivalis |
| *L. noguchi* | Australis | Bajan | Toad 60 | Barbados | Toad |
| *L. noguchi* | Australis | Peruviana | V 42 | Peru | Cattle |
| *L. santarosai* | Mini | Szwajizak | Szwajizak | Australia | Human |
| *L. santarosai* | Mini | Beye | 1537 U | Panama | Spiny rat |
| *L. santarosai* | Mini | Georgia | LT 117 | USA | Racoon |
| *L. santarosai* | Mini | Tabaquite | TRVL 3214 | Trinidad | Human |
| *L. santarosai* | Sejroe | Caribe | TRVL 61866 | Trinidad | Rat |
| *L. santarosai* | Sejroe | Gorgas | 1413 U | Panama | Spiny rat |
| *L. santarosai* | Sejroe | Trinidad | TRVL 34056 | Trinidad | Human |
| *L. santarosai* | Tarassovi | Atchafalaya | LSU 1013 | USA | Opossum |
| *L. santarosai* | Tarassovi | Atlantae | LT 81 | USA | Opossum |
| *L. santarosai* | Tarassovi | Bravo | Bravo | Panama | Human |
| *L. santarosai* | Tarassovi | Chagres | 1913 K | Panama | Spiny Rat |
| *L. santarosai* | Tarassovi | Darien | 637 K | Panama | Opossum |
| *L. santarosai* | Tarassovi | Navet | TRVL 109873 | Trinidad | Human |
| *L. santarosai* | Tarassovi | Rama | 316 | Nicaragua | Opossum |
| *L. santarosai* | Tarassovi | Sulzerae | LT 82 | USA | unknown |
| *L. santarosai* | undesignated | Peru | MW 10 | Peru | Opossum |
| *L. weilii* | Celledoni | Celledoni | Celledoni | Australia | Human |
